# *Streptococcus suis* autolysin functions as a molecular bridge for epithelial adhesion via lipoteichoic acid interaction

**DOI:** 10.1128/iai.00760-25

**Published:** 2026-05-13

**Authors:** Mingxing Liu, Hong Zhou, Xin Shan, Jingzhi Yuan, Kaiyue Yang, Mengzan Yang, Jinsheng Tang, Fei Pan, Huixing Lin, Zhe Ma, Hongjie Fan

**Affiliations:** 1 MOE Joint International Research Laboratory of Animal Health and Food Safety, College of Veterinary Medicine, Nanjing Agricultural University 70578, Nanjing, China; 2 Jiangsu Co-innovation Center for Prevention and Control of Important Animal Infectious Diseases and Zoonoses, Yangzhou, China; 3 College of Animal Science, Anhui Science and Technology University, Fengyang, China; University of Illinois Chicago, Chicago, Illinois, USA

**Keywords:** *Streptococcus suis*, autolysin, molecular bridge, adhesion, lipoteichoic acid

## Abstract

As a zoonotic opportunistic pathogen, adhesion to the porcine upper respiratory tract epithelium is a prerequisite for *Streptococcus suis* serotype 2 (SS2) to breach epithelial barriers and cause systemic infections under certain conditions. In this study, we first demonstrated the adhesive function of the autolysin Atl of SS2 to swine tracheal epithelial cells. We found that Atl functions as a molecular bridge for epithelial adhesion. The Bsp-like domain (Atl^Bsp^) specifically binds bacterial lipoteichoic acid, anchoring to the bacterial surface, while the C-terminal hydrolase domain (Atl^COOH^) directly interacts with host epithelial receptor Fibronectin (Fn). With the purified recombinant Atl (rAtl), we demonstrated that both membrane-bound and secreted forms of Atl could mediate adhesion to the epithelial cells. Moreover, the rAtl was able to restore the adhesive capacity of the Atl-deficient SS2 mutant (Δ*atl*) to both porcine and murine upper respiratory tract epithelium. This study reveals a novel function of the autolysin Atl in promoting SS2 adhesion to the upper respiratory tract epithelium, which elucidates the underlying molecular mechanism and identifies the key functional domains involved. These findings offer new insights into autolysin biology and provide a theoretical basis for developing anti-adhesion strategies against streptococcal infections in pigs.

## INTRODUCTION

*Streptococcus suis* (*S. suis*) infections and their transmission among pigs are widespread globally, particularly in major pig-raising countries, where they pose a serious threat to the swine industry (1). There are 29 serotypes of *S. suis*, with serotypes 2, 7, and 9 exhibiting high virulence (2–4). Respiratory tract colonization is a critical initial step in SS2 infection, serving not only as a foundation for its long-term survival within the host but also as a key prerequisite for secondary systemic infections (5, 6). Furthermore, SS2 is a zoonotic pathogen that can infect humans through skin wounds, particularly in individuals with occupational exposure to pigs or pork products. Once in the bloodstream, it can cross the blood-brain barrier and cause meningitis, which is the most common clinical manifestation in human cases (7, 8). Currently, *S. suis* has raised several public health concerns due to infections in pigs caused by SS2 (9, 10).

To adhere to epithelial cells, bacteria employ a variety of adhesion strategies. During infection, bacteria extend their adhesion proteins outward to facilitate the binding between pathogen adhesion ligands and receptors, thereby promoting adhesion (11). Bacteria tend to adhere to epithelial cells following the viral upregulation of bacterial receptors (12, 13). Some bacteria enhance adhesion by binding to secreted proteins that interact with epithelial cells (14).

Autolysin, a kind of cell wall hydrolase, plays a crucial role in bacterial division. To mitigate excessive damage to the cell wall caused by the autolysin cell wall hydrolase domain, gram-positive bacteria preserve the cell wall hydrolase on membrane-bound lipoteichoic acid (LTA) and wall-bound teichoic acid (WTA) with highly repetitive Bsp-like domains (15). The inhibition or deletion of lipoteichoic acid synthetase (LtaS) results in lipoteichoic acid (LTA) depletion, mislocalization of hydrolase, and subsequent damage to the bacterial cell wall (16–19). The structure of SS2 Atl, which contains six highly repetitive Bsp-like domains (Atl^Bsp^) and a C-terminal cell wall hydrolase domain (Atl^COOH^), is schematically represented in Fig. S1. Following the knockout of *ltaS* in SS2, significant damage to the SS2 cell wall was also observed (20). Except for inducing bacterial division, Atl was identified as a key adhesion protein (21, 22). Our previous research indicated that Atl enhances SS2 adhesion to swine tracheal epithelial cells (STECs) (21). However, the specific mechanism by which SS2 utilizes Atl to promote its adhesion to epithelial cells during early infection remains unclear.

Our study demonstrates that Atl interacts with LTA and is located on the SS2 surface through Atl^Bsp^. Moreover, the carboxy-terminal hydrolase domain of Atl (Atl^COOH^) functions as the ligand domain, facilitating SS2 adhesion to respiratory epithelial cells in the upper respiratory tract.

## RESULTS

### LTA deficiency decreases adhesion protein Atl level on SS2

**Fig 1:**
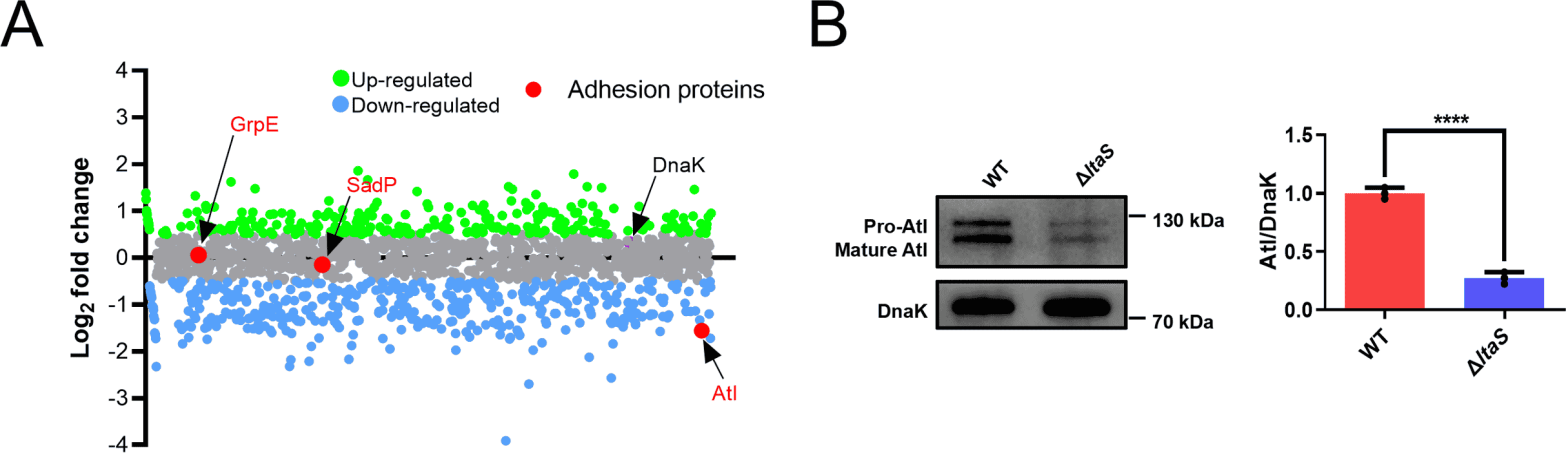
LtaS regulates Atl level on SS2. (**A**) Distribution of bacterial protein ratios for the total proteins quantified in WT SS2 and Δ*ltaS*. Compared with WT SS2, 256 proteins were upregulated (log_2_ [ratio (Δ*ltaS*/WT)] > 0.5, green) and 402 were downregulated (log_2_ [ratio (Δ*ltaS*/WT) <−0.5, blue]) in Δ*ltaS*. Adhesion proteins were tagged with red. (**B**) Validation of protein Atl quantification by Western blot assay. DnaK was used as a loading control. Quantification of Atl is shown on the right. Data shown are means ± SD; ****P < 0.0001 (two-tailed Student’s *t*-tests).

LtaS is a membrane-anchored enzyme that processively polymerizes the glycerolphosphate backbone of LTA on the extracellular side of the membrane, using phosphatidylglycerol as the substrate donor (23). LTA has been identified as a membrane anchor for the autolysin AtlA in *Staphylococcus aureus*, mediating its adherence to host cells (24, 25). SS2 autolysin Atl was previously reported to mediate attachment to host cells (21). To determine whether the SS2 autolysin Atl similarly interacts with LTA, we constructed an LTA-deficient SS2 mutant (Δ*ltaS*) (Fig. S2). To investigate whether the loss of LtaS broadly affects the abundance of surface-associated proteins, we performed a comparative proteomic analysis of wild-type (WT) SS2 and the Δ*ltaS* mutant. Protein abundance was normalized using DnaK as an internal reference, as its levels remained stable between the two strains (−0.5 < log_2_ [ratio (Δ*ltaS*/WT) < 0.5]) (14). The comprehensive data of proteomics analysis revealed that the adhesins GrpE and SadP were unchanged in the Δ*ltaS* mutant compared to WT SS2 (−0.5 < log_2_ [ratio (Δ*ltaS*/WT) < 0.5]) (26, 27). However, the level of Atl was markedly reduced (log_2_ [ratio (Δ*ltaS*/WT) <−0.5]) (Fig. 1A), indicating that LTA is specifically required for maintaining normal Atl levels. As shown in Fig. 1B, cell lysates collected at the logarithmic phase revealed reduced levels of both mature Atl and its precursor pro-Atl in the Δ*ltaS* mutant compared to WT SS2. These results suggest that LTA deficiency decreases adhesion protein Atl level on SS2.

### Atl interacts with SS2

**Fig 2:**
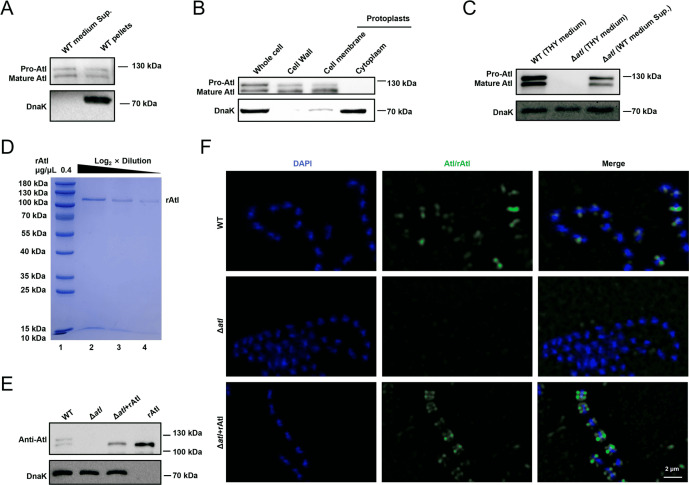
Atl interacts with SS2. (**A**) The subcellular distribution of Atl and DnaK in the medium and bacteria from WT SS2 was determined by Western blot. (**B**) The subcellular distribution of Atl and DnaK in the cell wall, cell membrane, and cytoplasm from WT SS2 was determined by Western blot. (**C**) The distribution of Atl in the bacteria from WT SS2, Δ*atl*, and Δ*atl* with the addition of WT SS2 culture supernatant was determined by Western blot. DnaK was used as a loading control. (**D**) Purified recombinant Atl (rAtl) expressed in *E. coli* was analyzed using SDS-PAGE and stained with Coomassie Brilliant Blue R-250. Lane 1, protein marker; Lanes 2–4, purified rAtl. (**E**) The distribution of Atl in the bacteria from WT SS2, Δ*atl*, and Δ*atl* supplemented with rAtl was determined by Western blot. DnaK was used as a loading control. (**F**) Confocal images of bacteria. WT SS2, Δ*atl*, and Δ*atl* supplemented with rAtl were subsequently labeled with anti-Atl polyclonal antibody and Alexa Fluor 488-conjugated anti-rabbit antibody (green), and nuclei of SS2 were stained with DAPI (blue). Scale bar, 2 μm.

To explore the distribution of Atl and DnaK in SS2, we extracted SS2 proteins from the medium, cell wall, cell membrane, and cytoplasm. Western blot analyses determined that Atl could be detected in the culture supernatant, cell wall, and cell membrane, and DnaK could be detected in the protoplasts (Fig. 2A and B).

Bacteria secrete proteins through specialized secretory systems, and secreted proteins can subsequently be anchored to the cell surface or recycled (14, 28). Given that Atl induces SS2 autolysis (Fig. S3A). To explore whether Atl interacted with the Δ*atl* mutant and induced autolysis, the Δ*atl* strain was cultured in WT SS2 culture supernatant. As shown in Fig. 2C, Atl was found to interact with the Δ*atl* mutant. Then the autolytic activities of WT SS2, Δ*atl*, and Δ*atl* with the addition of WT SS2 culture supernatant were measured. The result indicated that WT SS2 OD_600_ value decreased within 8 hours, and Δ*atl* had no autolysis after 8 hours of incubation, while the OD_600_ value of Δ*atl* with the addition of WT SS2 culture supernatant decreased (Fig. S3B). Therefore, Atl in WT SS2 culture supernatant interacted with Δ*atl* and induced Δ*atl* autolysis.

To explore the interaction between Atl and SS2 *in vitro*, the expression plasmid pColdI-*atl* was transformed into *E. coli* BL21 (DE3). *E. coli* BL21 (DE3) overproduced the rAtl under the induction of IPTG, and rAtl was purified with a Ni-affinity column. The corresponding purified fusion protein His-Atl was detected by SDS-PAGE (Fig. 2D). As shown in Fig. 2E, Western blot did not detect Atl in the Δ*atl*, while Atl was detected in the Δ*atl* supplemented with recombinant Atl. Given that Atl is distributed on both the cell wall and cell membrane of SS2 (Fig. 2B), further indirect immunofluorescence (IFA) analysis demonstrated its predominant localization at the bacterial surface division septum, while fluorescence was also noticed in the Δ*atl* division septum location with the addition of rAtl (Fig. 2F). Then, the autolytic activities of WT SS2, Δ*atl*, and Δ*atl* supplemented with rAtl were measured. As shown in Fig. S3C, the OD_600_ value of WT SS2 and Δ*atl* supplemented with rAtl decreased. The results indicated that the addition of rAtl restored autolysis in the Δ*atl* mutant. These results suggest that the Atl protein may directly interact with some surface factors of SS2.

### Atl is localized on the membrane of SS2 by interacting with LTA

**Fig 3:**
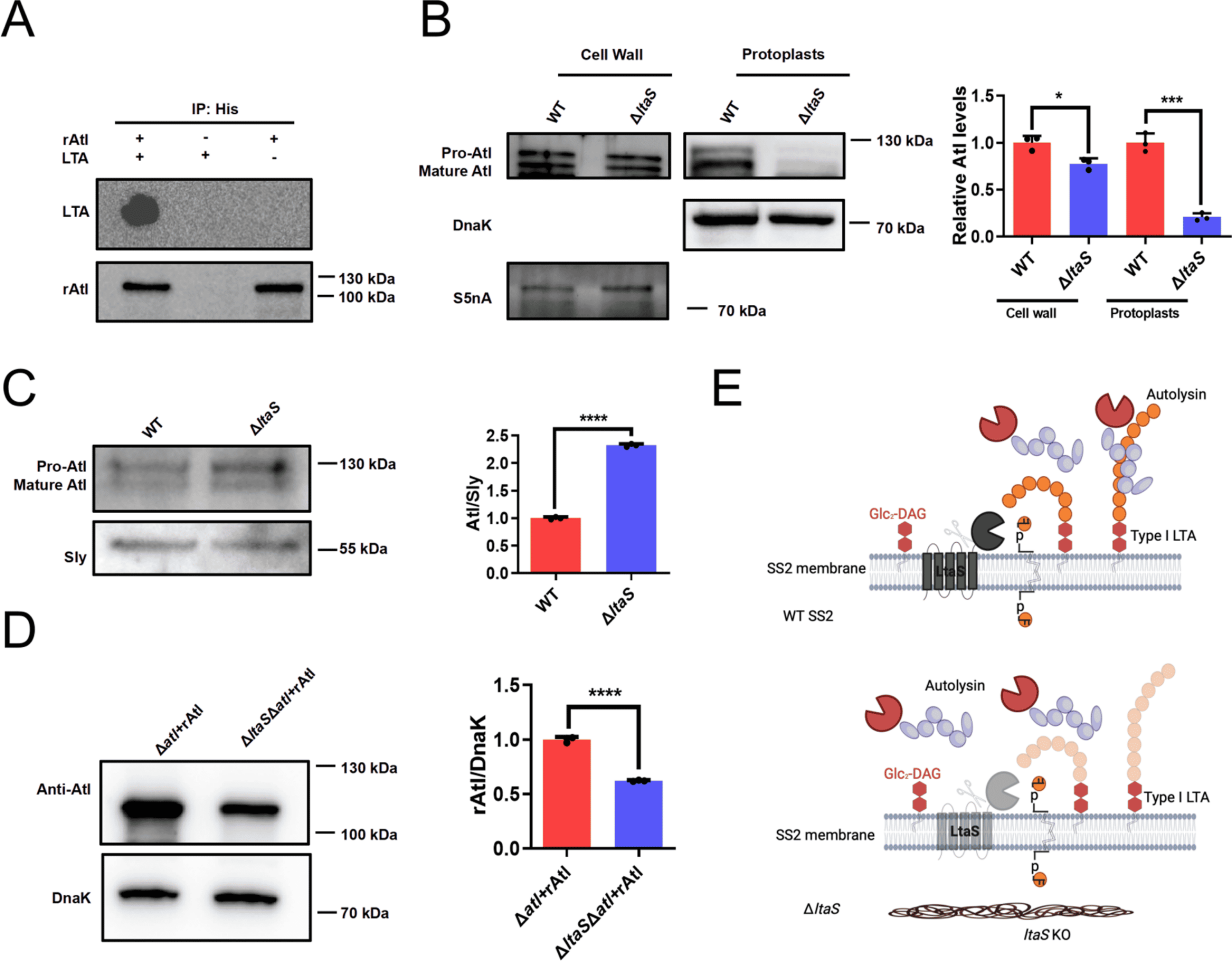
LTA interacting with Atl promotes SS2 combining with Atl. (**A**) Pull-down assay to determine the interaction between Atl and LTA. His-tagged Atl expressed in *E. coli* (DE3) strains was conjugated to Ni-affinity beads. Ni-affinity beads with His-tagged Atl and empty Ni-affinity beads were incubated with LTA. After being washed, the samples were analyzed by immunoblotting with anti-LTA mAb and anti-Atl polyclonal antibody. (**B**) The subcellular distribution of Atl in the cell wall and cell membrane from WT SS2 and Δ*ltaS* was determined by Western blot. DnaK was used as a protoplast loading control, and S5nA was used as a cell wall loading control. Quantification of Atl in WT SS2 and Δ*ltaS* is shown on the right. Data shown are means ± SD; *P < 0.05; ***P < 0.001 (two-tailed Student’s *t*-tests). (**C**) WT SS2 and Δ*ltaS* were cultured in THY medium, and the medium was subsequently collected. The presence of Atl in the medium was confirmed through Western blot analysis. Sly was used as a loading control. Quantification of Atl in WT SS2 medium and Δ*ltaS* medium is shown on the right. Data shown are means ± SD; ****P < 0.0001 (two-tailed Student’s *t*-tests). (**D**) Protein Atl in Δ*ltaS*, Δ*atl*, and Δ*ltaS*Δ*atl* was determined through Western blot analysis. DnaK was used as a loading control. Quantification of Atl in WT SS2 and Δ*ltaS* is shown on the right. Data shown are means ± SD; ****P < 0.0001 (two-tailed Student’s *t*-tests). (**E**) A schematic represents the mechanism of LTA regulating Atl anchoring on SS2. After SS2 *ltaS* is knocked out, the skeletal structure of LTA is lost, and the autolysin anchoring on LTA is secreted to the medium (created with BioRender.com).

Previous studies have shown that secreted bacterial proteins can interact with WTA and localize on the cell surface. In the absence of WTA galactosylation, these proteins fail to anchor onto the bacterial surface and are instead released into the surrounding environment (15). SS2 has the typical type I LTA and mixed-type series of LTA (29, 30). Given that LTA deficiency decreases Atl level on SS2 (Fig. 1), we hypothesized that LTA plays a crucial role in Atl surface display. To investigate whether LTA directly binds to Atl and functions as an anchoring molecule, we performed pull-down assays. As shown in Fig. 3A, in the pull-down assay, type I LTA was successfully captured by rAtl, indicating a direct interaction between the two molecules. Given that Atl is not present in the cytoplasm (Fig. 2B), the protoplast-associated Atl is localized to the membrane, and DnaK can be the loading control for SS2 cell membrane Atl. To further investigate the relevance of Atl localization with SS2 LTA *in vivo*, we separately extracted cell wall and protoplast-associated proteins from WT and Δ*ltaS* and assessed the distribution of Atl. We selected Suilysin (Sly) and Streptococcal 5′-Nucleotidase A (S5nA), respectively, as the loading controls for SS2 secreted proteins and cell wall proteins (31, 32). Interestingly, the levels of Atl in the cell wall fraction were comparable between WT and Δ*ltaS* strains, whereas their levels were markedly reduced in the protoplast fraction of the Δ*ltaS* mutant (Fig. 3B), while Western blot analysis of the culture supernatant revealed that the Δ*ltaS* released significantly higher amounts of Atl than the WT strain (Fig. 3C), suggesting that the absence of LTA anchoring may lead to increased release of Atl. Furthermore, deletion of the *ltaS* gene in the Δ*atl* background (Δ*ltaS*Δ*atl* double mutant) slightly impaired the strain’s ability to combine rAtl (Fig. 3D). Collectively, these results indicate that LTA plays a key role in Atl anchoring on the SS2 membrane (Fig. 3E).

### Atl functions as a molecular bridge for SS2 adhesion

**Fig 4:**
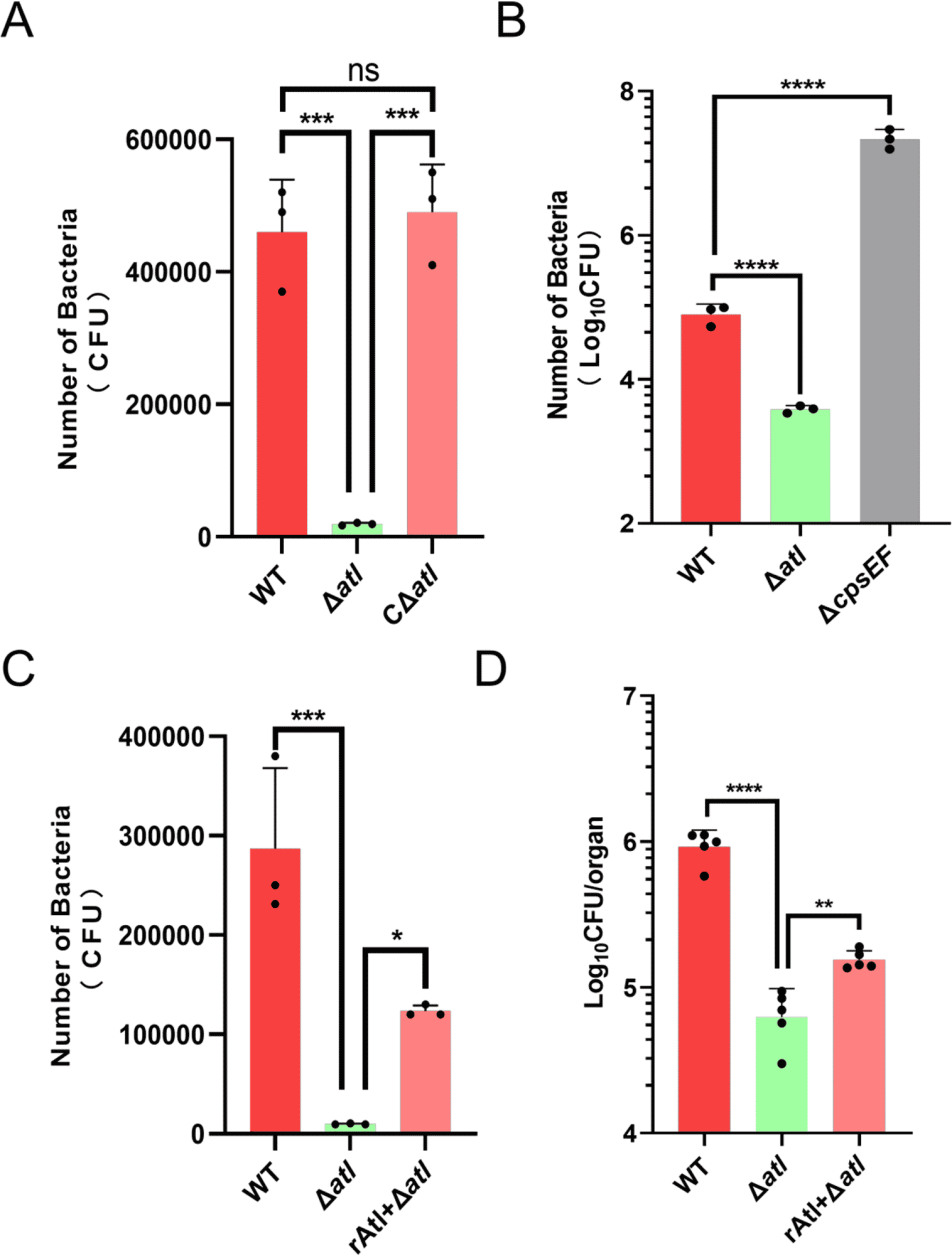
Atl enhances the adhesion of SS2 to STEC and the upper respiratory tract of mice. (**A**) Adhesion of SS2 to STEC. The cells were infected with WT SS2 (MOI = 100), Δ*atl* (MOI = 100), or CΔ*atl* (MOI = 100) for 2 hours. The number of adherent bacteria was counted. The CFU of SS2 is shown. Data are presented as means ± SD. ***P < 0.001; ns, not significant (one-way ANOVA). (**B**) Adhesion of SS2 to STEC. The cells were infected with WT SS2 (MOI = 100), Δ*atl* (MOI = 100), or Δ*cpsEF* (MOI = 100) for 2 hours. The number of adherent bacteria was counted. The Log_10_CFU of SS2 is shown. Data are presented as means ± SD. ****P < 0.0001 (one-way ANOVA). (**C**) Adhesion of SS2 to STEC. The cells were infected with WT SS2 (MOI = 100) or Δ*atl* (MOI = 100) for 2 hours. STECs were pre-treated with 10 μg rAtl for 2 hours, and the cells were infected with Δ*atl* (MOI = 100) for another 2 hours. The number of adherent bacteria was counted. The CFU of SS2 is shown. Data are presented as means ± SD. *P < 0.05; ***P < 0.001 (one-way ANOVA). (**D**) Adhesion of SS2 to nasal cavities of mice. Nasal cavities of mice were infected with WT SS2 (2 × 10^9^) or Δ*atl* (2 × 10^9^) for 24 hours, and the number of bacteria in the nasal cavities of mice was counted. Nasal cavities of mice were pre-treated with PBS or 20 μg rAtl for 2 hours, the mice were infected with Δ*atl* (2 × 10^9^) for another 24 hours, and the number of bacteria in the nasal cavities of mice was counted. The CFU of SS2 is shown. Data are presented as means ± SD. **P < 0.01; ****P < 0.0001 (one-way ANOVA).

To determine the role of Atl in adhesion of SS2 to STEC, we assessed the adhesion abilities of the WT SS2, Δ*atl,* and the complemented strain CΔ*atl* using STEC. Analysis of adhesion abilities of WT SS2, Δ*atl*, and CΔ*atl* revealed that Δ*atl* exhibited impaired adhesion abilities in comparison with the WT SS2, while complementation of *atl* restored Δ*atl* adhesion to epithelial cells (Fig. 4A). At the same time, there are no significant differences in growth curves among the three strains (Fig. S4A and B), indicating that the adhesion defect of the Δ*atl* mutant is not attributable to impaired growth. These findings establish that Atl mediates SS2 adhesion to epithelial cells.

The thickness of the bacterial capsule affects its adhesion to epithelial cells (33–35). To exclude the influence of capsule thickness on bacterial adhesion, thereby allowing us to specifically assess the role of SS2 Atl, we constructed a *cpsEF*-deficient mutant (Δ*cpsEF*) as previously described (36). We assessed the adhesion abilities of the WT SS2 and Δ*cpsEF* using STEC. Similarly, the adhesion of Δ*cpsEF* was higher than that of the WT strain (Fig. 4B). We analyzed the capsule thickness of WT SS2, Δ*atl*, and Δ*cpsEF* by TEM. The results showed that compared with WT SS2, the capsule of Δ*cpsEF* was missing, while the *atl* deletion did not affect the thickness of the SS2 capsule (Fig. S5). Therefore, we excluded the possibility that Atl affects its adhesion to STEC by regulating the capsular thickness.

Given that Atl localized on the bacterial surface promotes the adhesion of SS2 to STEC (Fig. 2B and 4A). However, it remains unknown whether secreted Atl can act as a bridge by binding bacteria and STEC, thereby enhancing SS2 adhesion. We hypothesized that the interaction between Atl and SS2 makes a contribution to the adhesion of SS2 to the STEC. To determine whether the defect in adhesion could be rescued by exogenous rAtl, we pre-treated STEC with rAtl prior to infection with the Δ*atl* mutant. Adherence assays comparing WT SS2 and the Δ*atl* mutant to STEC indicated that pretreatment with rAtl significantly increased the adhesion of the Δ*atl* mutant to STEC without affecting bacterial growth (Fig. 4C; Fig. S4C and D), indicating that Atl interacting with STEC plays an important role in increasing SS2 adhesion. Adherence assays of the WT SS2 and Δ*atl* to nasal cavities of mice indicated that pretreatment of rAtl increased Δ*atl* adhesion in the murine upper respiratory tract (Fig. 4D), respectively, again confirming the importance of the interaction between Atl and SS2 in adhesion of SS2 to the upper respiratory tract epithelial cells *in vivo*.

### Atl^Bsp^ interacts with LTA

**Fig 5:**
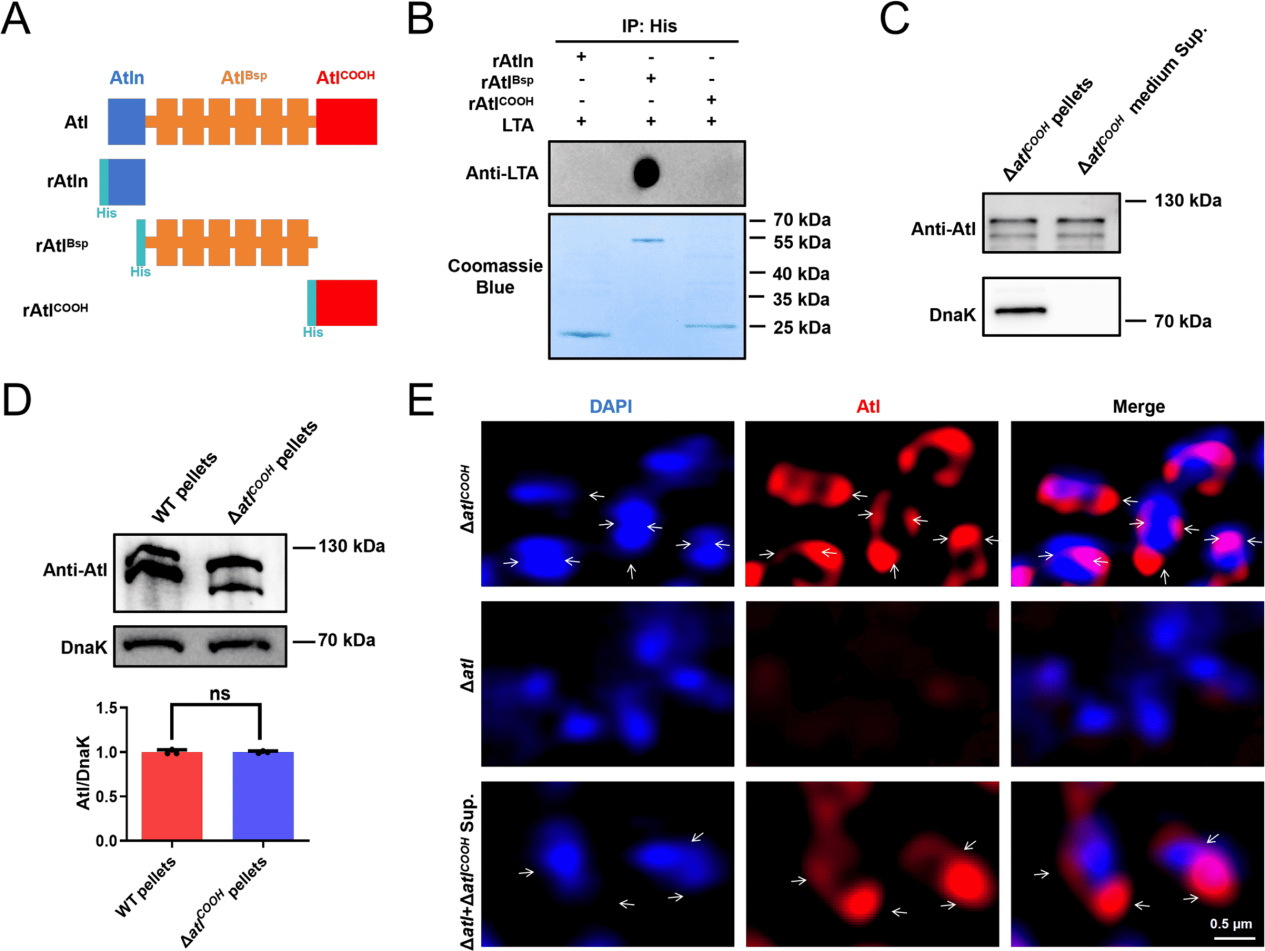
SS2 interacts with LTA through its Atl^Bsp^ domain. (**A**) A schematic representation of the SS2 Atl and rAtl domains (created with BioRender.com). (**B**) Pull-down assay determined the interaction between Atl domains and LTA. His-tagged Atl domains expressed in *E. coli* (DE3) strains were conjugated to Ni-affinity beads and incubated with LTA. After being washed, the bound protein-LTA complex was analyzed by immunoblotting with anti-LTA mAb and Coomassie Brilliant Blue R-250 staining. (**C**) The subcellular distribution of Atl in the medium and bacteria from Δ*atl^COOH^* was determined by Western blot. (**D**) The presence of Atl in the SS2 was confirmed through Western blot analysis. DnaK was used as a loading control. Quantification of Atl in WT SS2 medium and Δ*ltaS* medium is shown. Data shown are means ± SD; ns, not significant (two-tailed Student’s *t*-tests). (**E**) Confocal images of bacteria. The Δ*atl^COOH^* labeled by protein fusions. Δ*atl^COOH^*, Δ*atl,* and Δ*atl* with the addition of Δ*atl^COOH^* culture supernatant were examined by fluorescence microscopy. The white arrow indicates Atl labeled by mCherry (red), and nuclei of SS2 were stained with DAPI (blue). Scale bar, 0.5 μm.

The *S. suis* autolysin Atl comprises three functional domains: N-terminal domain (Atln), Atl^Bsp^, and Atl^COOH^. To identify the domain of Atl responsible for interacting with LTA, recombinant His-tagged Atl domains: rAtln, rAtl^Bsp^, and rAtl^COOH^ were designed for reacting with LTA (Fig. 5A). To confirm the interaction between LTA and the candidate Atl domains, LTA interacted with His-tagged Atl domains separately. As shown in Fig. 5B, LTA was pulled down with Atl^Bsp^. While LTA was not pulled down with Atln and Atl^COOH^. This suggests that the Bsp domain of Atl interacts with LTA. To further confirm the direct interaction between LTA and Atl^Bsp^ *in vivo*, we constructed SS2 Atl fused mutant *atl^～mCherry^* and truncated mutant Δ*atl^COOH^* in the WT SS2 background. As shown in Fig. S6, Atl can be labeled by protein fusions and distributed in the division septum location. Western blot results showed that Atl truncate was detected in the culture supernatant of the Δ*atl^COOH^* mutant, indicating that deletion of the COOH domain does not affect Atl secretion (Fig. 5C). Meanwhile, the levels of Atl were comparable between WT and Δ*atl^COOH^* strains, indicating that expression of the Atl truncate in Δ*atl^COOH^* was not degraded and expressed at the same level as the WT SS2 (Fig. 5D). To further confirm the interaction of Atl^Bsp^ and LTA contributing to the interaction of secreted Atl with SS2, Δ*atl* was cultured in Δ*atl^COOH^* culture supernatant. Indirect immunofluorescence analysis detected Atl labeled by mCherry was noticed in the Δ*atl* division septum location with the addition of Δ*atl^COOH^* culture supernatant (Fig. 5E). Taken together, these data indicate that Atl^Bsp^ interacts with LTA and is involved in the proper localization of Atl on the SS2 surface.

### Atl^COOH^ interacting with STEC promotes SS2 adhesion to epithelial cells

**Fig 6:**
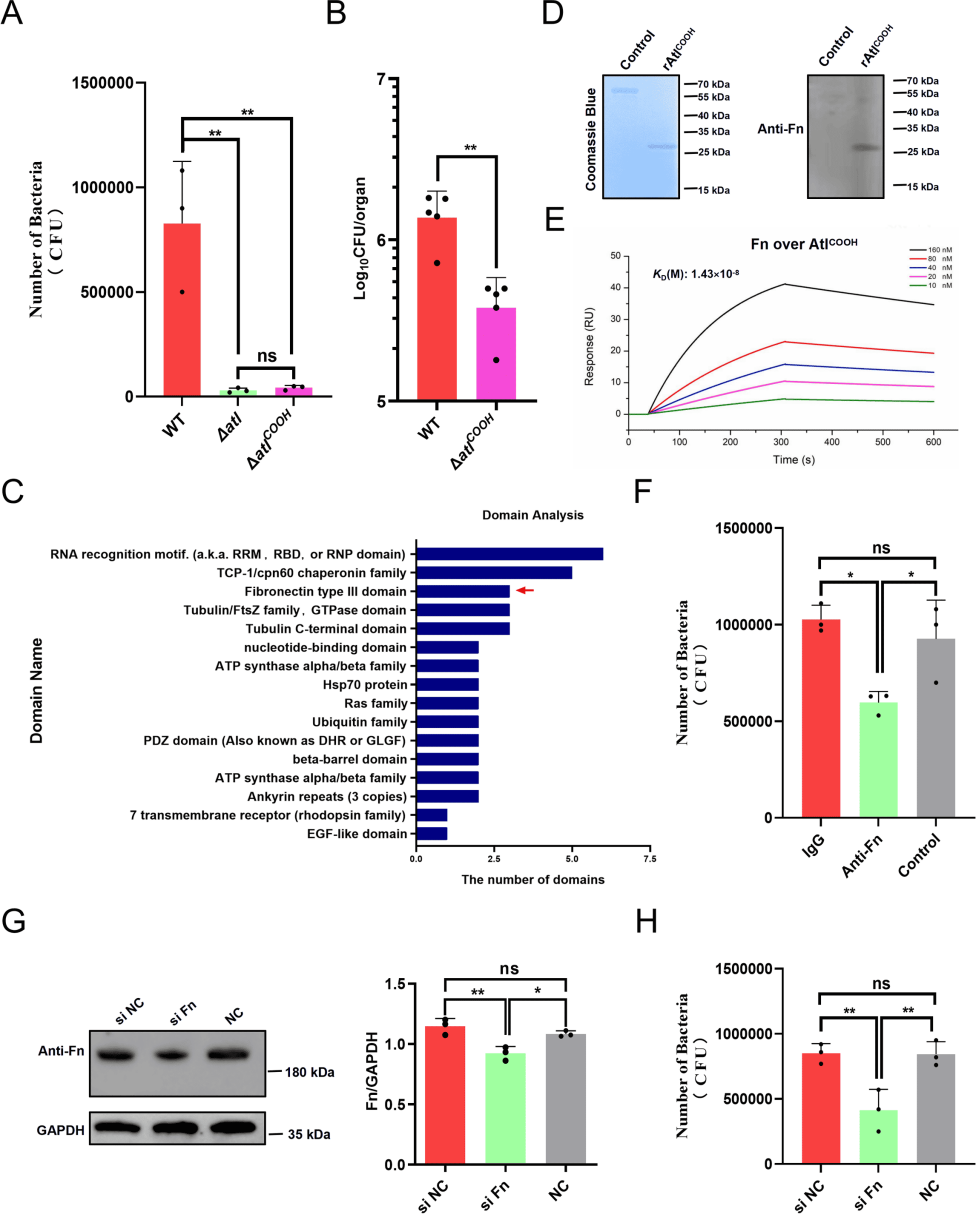
Fn is one of Atl^COOH^ receptors facilitating the adhesion of SS2 to epithelial cells. (**A**) Adhesion of SS2 to STEC. The cells were infected with WT SS2 (MOI = 100), Δ*atl* (MOI = 100), or Δ*atl^COOH^* (MOI = 100) for 2 hours, and the number of adherent bacteria was counted. The CFU of SS2 are shown. Data are presented as means ± SD. **P < 0.01; ns, not significant (one-way ANOVA). (**B**) Adhesion of SS2 to nasal cavities of mice. The nasal cavities of mice were treated with WT SS2 (2 × 10^9^) or Δ*atl^COOH^* (2 × 10^9^) for 24 hours, and the number of bacteria in the nasal cavities of mice was counted. The CFU of SS2 is shown. Data are presented as means ± SD. **P < 0.01 (two-tailed Student’s t-tests). (**C**) Domains of murine respiratory epithelial cell protein interact with rAtl^COOH^. His-tagged Atl^COOH^ expressed in *E. coli* (DE3) strains was conjugated to Ni-affinity beads. Ni-affinity beads with His-tagged Atl^COOH^ were incubated with murine respiratory epithelial cell proteins. After being washed, the bound protein complex was subjected to LC-MS/MS. Domains of murine respiratory epithelial cell protein interacting with rAtl^COOH^ were presented. (**D**) The interaction of rAtl^COOH^ with Fn. rAtl^COOH^ was electrophoresed on a 12% SDS-PAGE gel and stained with Coomassie Brilliant Blue R-250. Electrophoresed proteins were transferred onto a PVDF membrane, then subjected to Far-Western blot analysis using Fn and anti-Fn polyclonal antibody. rAtl^Bsp^ was set as His-tagged control. (**E**) Surface plasmon resonance (SPR) experiment showing the dose-dependent binding profile of Fn (10–160 nM) over immobilized rAtl^COOH^. RU, response units. (**F**) Adhesion of SS2 to STEC. Before infection, STEC was treated with rabbit polyclonal anti-Fn antibody or rabbit immunoglobulin G (IgG) for 4 hours, followed by WT SS2 (MOI = 100) infection for another 2 hours, and the number of adherent bacteria was counted. The CFU of SS2 is shown. Data are presented as means ± SD. *P < 0.05; ns, not significant (one-way ANOVA). (**G**) Western blot analysis of Fn protein levels. STEC was transfected with NC siRNA or Fn siRNA for 12 hours. GAPDH was used as a loading control. Quantification of Fn in STEC is shown on the right. Data shown are means ± SD; **P < 0.01; *P < 0.05; ns, not significant (one-way ANOVA). (**H**) Adhesion of SS2 to STEC. STEC was transfected with NC siRNA or Fn siRNA for 12 hours. STEC was infected with WT SS2 (MOI = 100) for 2 hours, and the number of adherent bacteria was counted. The CFU of SS2 is shown. Data are presented as means ± SD. **P < 0.01; ns, not significant (one-way ANOVA).

It has been shown that the C-terminal catalytic domain of *Clostridium perfringens* (*C. perfringens*) and *Streptococcus mutants* (*S. mutans*) autolysin promotes bacterial adhesion (37, 38). These findings prompted us to examine whether Atl^COOH^ promotes SS2 adhesion. To determine the role of Atl^COOH^ in adhesion of STEC, we assessed the adhesion abilities of the WT SS2 and Δ*atl^COOH^* using STEC. Given that expression of the Atl truncated in Δ*atl^COOH^* was expressed at the same level as the WT SS2 (Fig. 5D), Δ*atl^COOH^* exhibited impaired adhesion abilities in comparison with the WT SS2 (Fig. 6A), indicating that Atl^COOH^ plays an important role in SS2 adhesion of respiratory epithelial cells. Adherence assays of the WT SS2 and Δ*atl^COOH^* to nasal cavities of mice confirmed the importance of Atl^COOH^ in adhesion of SS2 to the upper respiratory tract epithelial cells *in vivo* (Fig. 6B).

**Fig 7:**
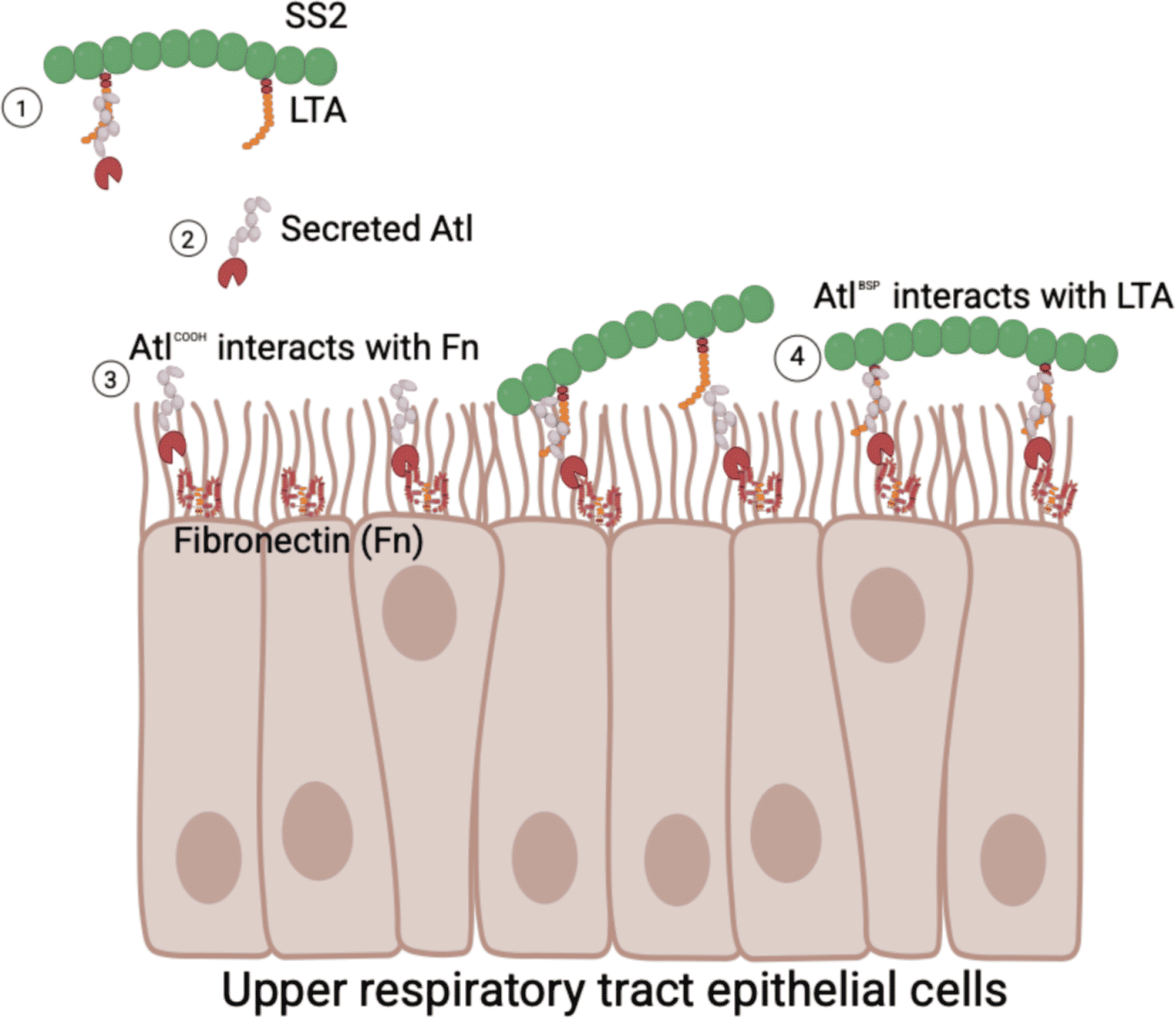
Diagram illustrating the mechanism by which Atl functions as a molecular bridge to promote efficient SS2 adhesion. SS2, which is breathed in the upper respiratory tract, secretes Atl (1 and 2). These secreted Atl molecules bind to Fn by Atl^COOH^ (3). Interaction between the Atl^Bsp^ domain and LTA at the cell membrane of SS2 promotes SS2 adhesion to the upper respiratory tract epithelial cells (4). A number of Atl molecules on the cell surface are utilized for the attachment of another SS2 (created with BioRender.com).

Given the role of Atl^COOH^ in the adhesion of SS2 in epithelial cells, we speculated that Atl^COOH^ mediates the adhesion of SS2 in epithelial cells by binding to cell surface receptors. A pull-down assay was performed after incubation of mice respiratory epithelial cell lysates with His-tagged Atl^COOH^ (rAtl^COOH^), and protein domains were analyzed. Pull-down assays revealed that Atl interacts with a spectrum of host proteins (Fig. 6C). Among these, the FnIII domain of fibronectin (Fn) was identified. To confirm the interaction of Atl^COOH^ with Fn on the surface of respiratory epithelial cells, a Far-Western blot was conducted. We found that Fn bound rAtl^COOH^ and did not bind His-tagged control protein (Fig. 6D). As shown in Fig. 6E, a SPR analysis showed the dissociation constant (KD) value between Atl^COOH^ and Fn was 1.43 × 10^−8^ M. Thus, Atl^COOH^ is interacted with Fn. To further validate the role of Fn in mediating Atl^COOH^-dependent bacterial adhesion, we performed antibody blockade assays and siRNA transfection. As shown in Fig. 6F through H, pretreatment of host cells with anti-Fn antibody or knocking down the expression of Fn decreased the adhesion of SS2. Together, these results showed that Atl^COOH^ interacting with STEC promotes SS2 adhesion to epithelial cells (Fig. 7).

## DISCUSSION

Atl was originally characterized as an autolysin required for daughter cell separation. Beyond its role in cell division, we previously demonstrated that Atl contributes to epithelial barrier disruption and facilitates bacterial invasion (21). These established functions position Atl as a key virulence factor that not only remodels the bacterial cell wall but also targets host cellular structures. In the present study, we identify a novel role for Atl as an adhesin mediating SS2 binding to epithelial cells. Complementation of the Δ*atl* mutant restored both adhesion and autolysis (Fig. 4A; Fig. S3A), indicating that Atl directly promotes host-pathogen interaction.

Unlike monolayer respiratory epithelial cells *in vitro*, the environment of upper respiratory epithelial cells is more complex. Especially, the mucus layer on the surface of the upper respiratory tract epithelial cells can effectively resist the adhesion of pathogens (39). During the transmission and infection of bacteria, the host also employs various mechanisms to eliminate pathogens from the surfaces of the upper respiratory and intestinal tracts (40, 41). However, pathogens can evade innate immune clearance by enhancing their adhesion to epithelial cells and forming biofilms (42, 43). The rapid adherence of pathogens to epithelial cells via adhesion proteins enhances colonization and infection (44–47). In the process of adherence, adhesion proteins play a crucial role in enhancing the attachment of bacteria to respiratory epithelial cells. This process not only promotes its adhesion in the upper respiratory tract but also inhibits the host’s clearance mechanisms. In this study, Atl has the function of promoting SS2 adhesion in both respiratory epithelial cells and the upper respiratory tract of mice. Therefore, Atl combined with respiratory epithelial cells mediates the adhesion of SS2 to the upper respiratory tract against the mucus layer.

We previously demonstrated that Atl^Bsp^ interacts with vimentin on STEC cells, and that deletion of *atl* in SS2 or knockout of vimentin in STEC significantly reduces bacterial adhesion (21). In the present study, we further identified that Atl^Bsp^ is also responsible for LTA binding on the bacterial surface (Fig. 5B). These findings initially suggested that Atl^Bsp^ might function as a critical adhesin by directly bridging SS2 to host vimentin. However, retention of Atl^Bsp^ did not enhance SS2 adhesion to STEC. This indicates that while Atl^Bsp^ can bind vimentin, this interaction is not sufficient to promote bacterial attachment. Instead, we found that Atl^COOH^ binds to Fn on the STEC surface, implicating Fn as the primary adhesin receptor. It has been reported that the state of Fn on epithelial cells is dynamically regulated by vimentin phosphorylation, which, in turn, mediates pathogen adhesion and spread (48). Based on this, we hypothesize that vimentin on STEC cells may function as a regulator of Fn conformation, promoting the transition of Fn from a soluble to an insoluble state. This conformational change could expose cryptic binding sites for Atl^COOH^ or enhance Fn’s ability to bridge bacteria and STEC, thereby facilitating SS2 adhesion.

Fibronectin (Fn) is a multifunctional glycoprotein widely distributed on epithelial cells and fibroblasts, where it serves as a common receptor for bacterial adhesins (49). Fn binds to host α5β1 integrin and is exploited by numerous gram-positive pathogens, including *Staphylococcus aureus* FnBPA, Group A *Streptococcus* Protein F, and *G. parasuis* RlpA, to establish infection (12, 50, 51). Structurally, Fn comprises three types of repeating units: FnI, FnII, and FnIII. While FnI and FnII are recognized as the primary adhesion receptor regions for most gram-positive bacteria (52). Additionally, FnIII has recently been identified as a binding site for cell wall hydrolases, such as those from *C. perfringens* (37, 53). SS2 binds to Fn through the cell wall hydrolase protein receptor region, thereby promoting SS2 adhesion (Fig. 6). While Atl^COOH^ binding might involve one or more Fn subdomains, further investigation using recombinant subdomains is needed to confirm this hypothesis.

Overall, we identified the domain of SS2 Atl that is responsible for adhesion to epithelial cells and demonstrated that the secretion of Atl promotes SS2 adhesion to the upper respiratory tract by binding to respiratory epithelial cells and SS2 LTA. This study provides evidence that SS2 adhesion to respiratory epithelial cells is mediated by LTA and highlights the role of Atl in enhancing this adhesion.

## MATERIALS AND METHODS

### Bacterial strains and cells

The WT SS2 (ZY05719) strain (GenBank accession: CP007497.1) was isolated from Ziyang, Sichuan, China, and is currently stored in our laboratory at Nanjing Agricultural University, Nanjing, China. The Δ*atl* mutant and the complemented strain (CΔ*atl*) used in this study were generated and stored in our laboratory (21). These strains were cultivated in THB broth (THB; Hopebio, HB0311-3) supplemented with 2% yeast extract (Oxoid, LP0021B). Swine tracheal epithelial cells (STEC; Affandi-e, x1204502), obtained from Affandi-e, were cultured in Dulbecco’s modified Eagle’s medium (DMEM; Gibco, C11995500BT) supplemented with 10% fetal bovine serum (FBS; Gibco, 10270106) at 37°C in an atmosphere containing 5% CO_2_.

### Structural modeling of the Atl protein

The amino acid sequence of the SS2 strain ZY05719 Atl (GenBank accession: EF563970.1) was derived through *in silico* translation of its nucleotide sequence. The three-dimensional structure prediction was conducted using SWISS-MODEL (https://swissmodel.expasy.org/interactive) in automated modeling mode. The AlphaFold model AF-A1YSC3-F1 was chosen as the primary template due to its high sequence identity with the target.

### Construction of SS2 mutant strains

To construct the SS2 mutant strain Δ*ltaS*, the upstream and downstream flanking regions of the *ltaS* gene were amplified from SS2 using specific primers, which are listed in Table S1. For the construction of the SS2 mutant strains *atl^~mCherry^*, Δ*atl^COOH^*, and Δ*atl^Bsp^*, the upstream and downstream flanking regions of the Atl gene were amplified from either SS2 or the plasmid pCMV-N-mCherry, utilizing the primers also listed in Table S1. The transformation of SS2 was performed using GNWGTWVEE, which was procured from Nanjing GenScrip. The construction of SS2 mutant strains followed the methodologies outlined in the references (54). The transformed WT SS2 was screened for resistance, and an appropriate sucrose concentration was employed to identify non-resistant strains. Finally, the *S. suis* gene deletion strains were verified through PCR or Western blot analysis.

### Scanning electron microscopy and transmission electron microscopy

Logarithmic *S. suis* was transferred to fresh THY medium at a ratio of 1: 100. After culturing *S. suis* to an OD_600_ of 0.6, the cells were centrifuged at 4,000 rpm for 5 minutes, washed, and suspended twice in PBS, with the final OD_600_ adjusted to 2.0. *S. suis* was fixed with glutaraldehyde (Sinopharm, 30092436) and sent to the electron microscope platform of the State Key Laboratory of Nanjing Agricultural University. Electron microscopic samples were prepared and scanned using a scanning electron microscope (SEM; HITACHI, Regulus8100) and a transmission electron microscope (HITACHI, HT7800). The capsule of bacteria was measured by Image-Pro Plus. The data were analyzed using GraphPad Prism 8.

### Recombinant protein purification

Recombinant protein containing an endogenous His tag was derived from the pColdI cloning vector (Takara, 3361) in *E. coli* BL21, utilizing a Ni-affinity column (Solarbio, P2010). *E. coli* was cultured in 500 mL LB medium supplemented with 100 μg/mL ampicillin at 37°C for 3 hours, followed by incubation at 16°C for 30 minutes. Induction was performed using 1 mM IPTG (BioFroxx, 1122GR001) at 16°C for 12 hours. After a total of 40 minutes of sonication, consisting of cycles of 5 seconds of sonication followed by 5 seconds of rest (Branson Sonifier), the supernatants were collected for purification. For the purification of the His-tagged recombinant protein, the supernatants were processed using a Ni-column with the appropriate buffer. The protein concentration was assessed using the Bradford assay and monitored through SDS-PAGE.

### Determination of the autolysis curve

At the logarithmic phase, WT medium was collected through centrifugation at 8,000 rpm for 10 minutes and further filtered through a 0.22 μm filter (Millipore, SLGP033RB) for culturing Δ*atl*. Purified rAtl was added to THY medium (10 μg/mL final concentration) and further filtered through a 0.22 μm filter for culturing Δ*atl*. According to the reference, SS2 was collected at the logarithmic phase. SS2 was washed with PBS twice and resuspended in Tris-HCl. The optical density (OD_600_) of SS2 was adjusted to 0.6 with a final concentration of 0.05% Triton X-100 (BioFroxx, 143,307) and incubated at 37°C with shaking at 180 rpm (22). The OD_600_ of SS2 was subsequently measured at 37°C.

### Bacterial cell wall, cell membrane, and cytoplasmic protein separation

*S. suis* was cultured to logarithmic phase in THY medium. *S. suis* cell wall, cell membrane, and cytoplasmic proteins were extracted using modified buffers according to methods described previously (55, 56). The cells were harvested and resuspended in buffer A (30 mM Tris-HCl, 3 mM MgCl_2_, 25% sucrose, 125 U/mL mutanolysin [Sigma, 55466-22-3], pH 7.4) for incubation at 37°C for 60 minutes. After incubation, the cell was centrifuged at 4°C and 8,000 rpm for 10 min. The cell wall proteins were in the supernatants, and the protoplasts were in the pellets. Protoplasts were resuspended in buffer B (30 mM Tris-HCl, 3 mM MgCl_2_, pH 7.4) for sonication, consisting of 10 cycles of 5 seconds of sonication followed by 10 seconds of rest. After sonication, the cell was centrifuged at 4°C and 19,000 rpm for 60 minutes. The cell cytoplasmic proteins were in the supernatants, and the cell membrane proteins were in the pellets. Cell membrane proteins were preserved in −80°C. Cell wall and cytoplasmic proteins in supernatants were precipitated with trichloroacetic acid (TCA, 15% final concentration) at 4°C for 30 minutes. After precipitation, the cell wall and cytoplasmic proteins were centrifuged at 4°C and 8,000 rpm for 10 minutes. The cell wall and cytoplasmic pellets were washed with ice-cold acetone twice and preserved in −80°C.

### Western blot

A 5× protein loading buffer was added to the sample, which was subsequently heated in a metal bath at 100°C for 10 minutes. SDS-PAGE was performed for protein separation at 80 V. Proteins were transferred from the gel to a polyvinylidene difluoride (PVDF) membrane (Millipore, ISEQ00010) using a semi-dry transfer method (Bio-Rad, 1703957). A 5% skim milk solution was employed for blocking. The primary antibodies, Atl (1: 500), Sly (1: 500), S5nA (1: 500), DnaK (1: 500), and GAPDH (1: 1,000, Cell Signaling Technology, 5174S) were incubated overnight at 4°C. The PVDF membrane was then washed three times with PBST. Subsequently, either a mouse secondary antibody (1: 2,000, Engibody, AT0098) or a rabbit secondary antibody (1: 2,000, Engibody, AT0097) was applied at room temperature for 2 hours, followed by three washes with PBST. Finally, an ECL chemiluminescence solution (Biosharp, BL523A) was added to the PVDF membrane for exposure (Bio-Rad, 1708280).

### Dot blot

Ni-affinity beads, with or without rAtl, were mixed with LTA (Sigma, 56411-57-5) or ddH_2_O and incubated overnight at 4°C. rAtl was eluted using a high concentration of imidazole and subsequently dripped onto a PVDF membrane. Following the application of imidazole, the PVDF membrane containing the eluted protein was air-dried. A 5% skim milk solution was used to block the PVDF membrane for 30 minutes, after which it was washed three times with PBST. The LTA primary antibody (1: 50, Hycult Biotech, HM2048) was then applied to the PVDF membrane and incubated at 4°C overnight. The membrane was subsequently washed three times with PBST. The mouse secondary antibody was applied and incubated at room temperature for 2 hours, followed by three washes with PBST. Finally, ECL color rendering was performed.

### Indirect immunofluorescence staining

*S. suis* was cultured to an OD_600_ of 0.6 in THY medium, either alone or supplemented with rAtl (10 μg/mL final concentration) that had been filtered through a 0.22 μm filter. The bacteria were then fixed with 4% paraformaldehyde for 1 h. Following fixation, samples were washed three times with PBS. An anti-Atl polyclonal antibody was incubated with the samples overnight at 4°C. Afterward, the samples were washed with PBS three times. Alexa Fluor 488-conjugated anti-rabbit antibody (Beyotime, A0562) was applied in the dark at 37°C for 1 h, followed by three additional washes with PBS. Finally, the microscopic slides were sealed with DAPI sealer (Biosharp, BL793B), and ultra-high-resolution fluorescence microscopy was employed for imaging.

### Bacterial growth kinetics assay

WT SS2 and Δ*atl* single colonies were selected from THY agar plates and subsequently inoculated into 2 mL of THY medium. Both WT SS2 and Δ*atl* were cultivated to mid-log phase at 37°C with shaking. The bacterial suspensions were diluted in either fresh THY medium or THY medium supplemented with rAtl to achieve an OD_600_ of 0.04. A volume of 200 μL of the standardized inoculum was transferred to 24-well plates. The plates were then incubated at 37°C with shaking at 180 rpm for 14 hours in a spectrophotometer-equipped incubator (BioTek Synergy H1). OD_600_ readings were recorded every 15 minutes, following background subtraction (blank: sterile THY). A volume of 4 mL of the standardized inoculum was incubated at 37°C with shaking at 180 rpm for 14 hours. The standardized inoculum of 100 μL was used to dilute and plate onto THY agar plates every 1 h. The THY agar plates were then incubated overnight at 37°C, and the colonies were counted. Growth curves were generated using GraphPad Prism 8.

### Bacterial adherence assay

STECs were cultured in monolayer cells supplemented with 10% fetal bovine serum in 12-well plates. The cells were pretreated with rAtl (10 μg/mL final concentration) or PBS for 2 hours, washed three times with PBS, and subsequently infected with SS2 at a multiplicity of infection (MOI) of 1: 100. Each experimental group consisted of three replicates. The samples were incubated at 37°C in a 5% CO_2_ incubator for 2 hours. Non-adherent bacteria were removed by washing the cells three times with PBS. To release adherent bacteria, the cells were lysed using 1 mL of distilled deionized water (ddH_2_O). After performing serial 10-fold dilutions, 100 µL aliquots were plated on THY agar plates, and colonies were counted after incubation at 37°C.

### Mouse infection

Four- to six-week-old female C57BL/6J mice were obtained from Sikebas Biotechnology Co., Ltd. (Henan, China). The mice were housed under specific-pathogen-free (SPF) conditions in individually ventilated cages (IVCs) at the Laboratory Animal Center of Nanjing Agricultural University. Nasal cavities of mice were on pretreatment, pre-treated with PBS for 2 hours, or pre-treated with 20 μg rAtl (10 µg per nostril) for 2 hours. A 20 µL inoculum, containing 2 × 10^9^ CFU, was administered dropwise into each nostril (10 µL per nostril) using a micropipette. The mice were held upright for 30 seconds post-inoculation to ensure adequate bacterial deposition in the upper respiratory tract. At 24 hours post-infection, the mice were euthanized, and the nasal cavities were aseptically collected. Subsequently, the tissues were homogenized in 1 mL of sterile PBS. The homogenates were serially diluted (10-fold) in PBS. Aliquots of 100 µL from each dilution were plated onto THY agar plates. The plates were incubated overnight at 37°C, and the colonies were manually counted. Statistical data were analyzed and presented using GraphPad Prism 8.

### Far-Western blot

Far-Western blot assay was carried out as reference (22). Purified proteins (rAtl^Bsp^ and rAtl^COOH^) were transferred electrophoretically onto PVDF membranes using a semi-dry transfer method. PVDF membranes were blocked in 5% bovine serum albumin (BSA, Solarbio, A8020) at 4°C overnight. 30 μg/mL Fibronectin (YEASEN, 40113ES03) was incubated with the PVDF membranes at 4°C overnight. The primary antibody, anti-Fn rabbit polyclonal antibody (1: 1,000, BOSTER, BA1772), was incubated 4 hours at room temperature. Rabbit secondary antibody (1: 2,000) was applied at room temperature for 2 hours. ECL chemiluminescence solution was added to the PVDF membrane for exposure.

### SPR analysis

The binding ability of Atl^COOH^ to Fn was determined by SPR analysis as described previously, with minor modifications (12, 57). The activator is prepared by mixing 400 mM EDC and 100 mM NHS immediately prior to injection. The chip is activated for 240 seconds with the mixture at a flow rate of 20 μL/min. Dilute Atl^COOH^ to 60 μg/mL in immobilization buffer, then inject to sample channel at a flow rate of 20 μL/min. The chip is deactivated by 1 M ethanolamine hydrochloride at a flow rate of 20 μL/min for 240 seconds. Fn is diluted with the same analyte buffer to six concentrations (160, 80, 40, 20, 10, and 0 nM). Fn is injected into the sample channel at a flow rate of 20 μL/min for an association phase of 240 seconds, followed by a 310-second dissociation. The association and dissociation processes are all handled in the analyte buffer. Repeat six cycles of analysis according to the analyte concentrations in ascending order.

### siRNA transfection

siRNA transfection was carried out as described previously (12). si-FN (sense: 5′-GCCCAAUUGAGUGCUUCAUTT-3′; antisense: 5′-AUGAAGCACUCAAUUGGGCTT-3′) and si-NC (sense: 5′-UUCUCCGAACGUGUCACGUTT-3′; antisense: 5′-ACGUGACACGUUCGGAGAATT-3′) were synthesized by Shanghai GenePharma and used to knock down the expression of Fn in STEC cells. When the STEC cell density reached 30%–50%, the diluted si-FN/si-NC and Lipofectamine 2000 (Invitrogen, 11668019) were incubated in serum-free DMEM for 15 minutes at room temperature, and the working concentration of si-FN1/si-NC was 50 nM for transfection for 12 hours. SS2 infection was then performed.

### Statistical analysis

All experiments were performed three times independently. Data were expressed as means ± SD, and GraphPad Prism 8 was used for one-way analysis of variance (ANOVA) and two-tailed Student’s *t*-tests. *P*-value < 0.05 is considered statistically significant.

## SUPPLEMENTAL MATERIAL

Supplemental material iai.00760-25-s0001.docx Fig. S1 to S6; Table S1.

ASM does not own the copyrights to Supplemental Material that may be linked to, or accessed through, an article. The authors have granted ASM a non-exclusive, world-wide license to publish the Supplemental Material files. Please contact the corresponding author directly for reuse.
