# Supplementary material for: Streptococcus suis autolysin functions as a molecular bridge for epithelial adhesion via lipoteichoic acid interaction
Source: Infect Immun. 2026 May 13;94(6):e00760-25. doi: 10.1128/iai.00760-25 (PMC13248702; doi:10.1128/iai.00760-25)
Supplement: Supplemental material — Fig. S1 to S6; Table S1. [file iai.00760-25-s0001.docx]

***Streptococcus suis* autolysin functions as a molecular bridge for epithelial adhesion via lipoteichoic acid interaction**

Mingxing Liu ^1^, Hong Zhou ^1^, Xin Shan ^1^, Jingzhi Yuan ^1^, Kaiyue Yang ^1^, Mengzan Yang ^1^, Jinsheng Tang ^1^, Fei Pan ^1^, Huixing Lin ^1^, Zhe Ma ^1*^, Hongjie Fan ^1,2,3*^

^1^ MOE Joint International Research Laboratory of Animal Health and Food Safety, College of Veterinary Medicine, Nanjing Agricultural University, Nanjing, China

^2^ Jiangsu Co-innovation Center for Prevention and Control of Important Animal Infectious Diseases and Zoonoses, Yangzhou, China

^3^ College of Animal Science, Anhui Science and Technology University, Fengyang, China

Corresponding author: Zhe Ma ^1*^, Hongjie Fan ^1,2,3*^

**Email:**

Zhe Ma: mazhe@njau.edu.cn, and Hongjie Fan: fhj@njau.edu.cn


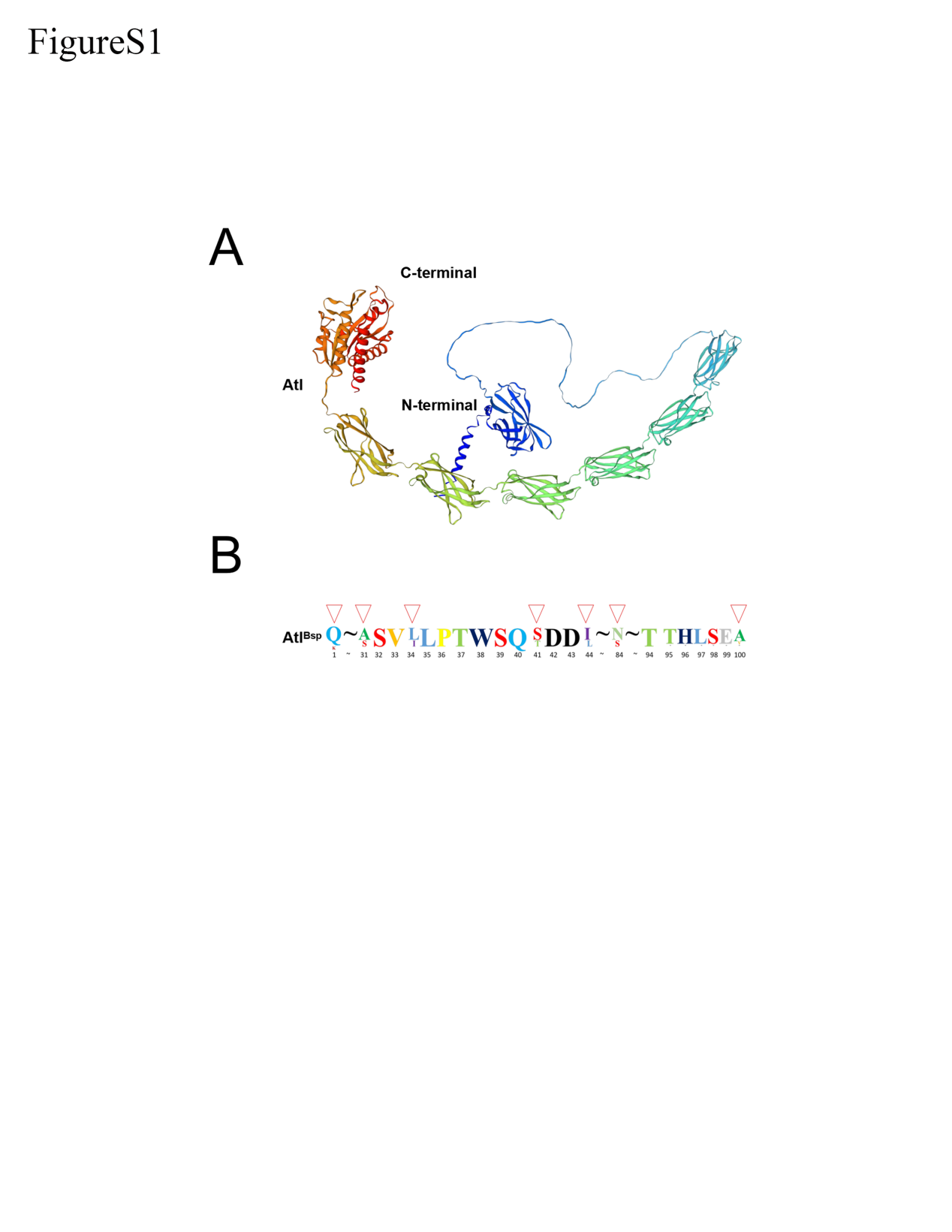


**Fig. S1** Schematic depiction of ZY05719 Atl and Atl^Bsp^ repeat sequence. Red triangles mark different amino acid in Bsp sequence. (created with swiss-model.com and BioRender.com).

**
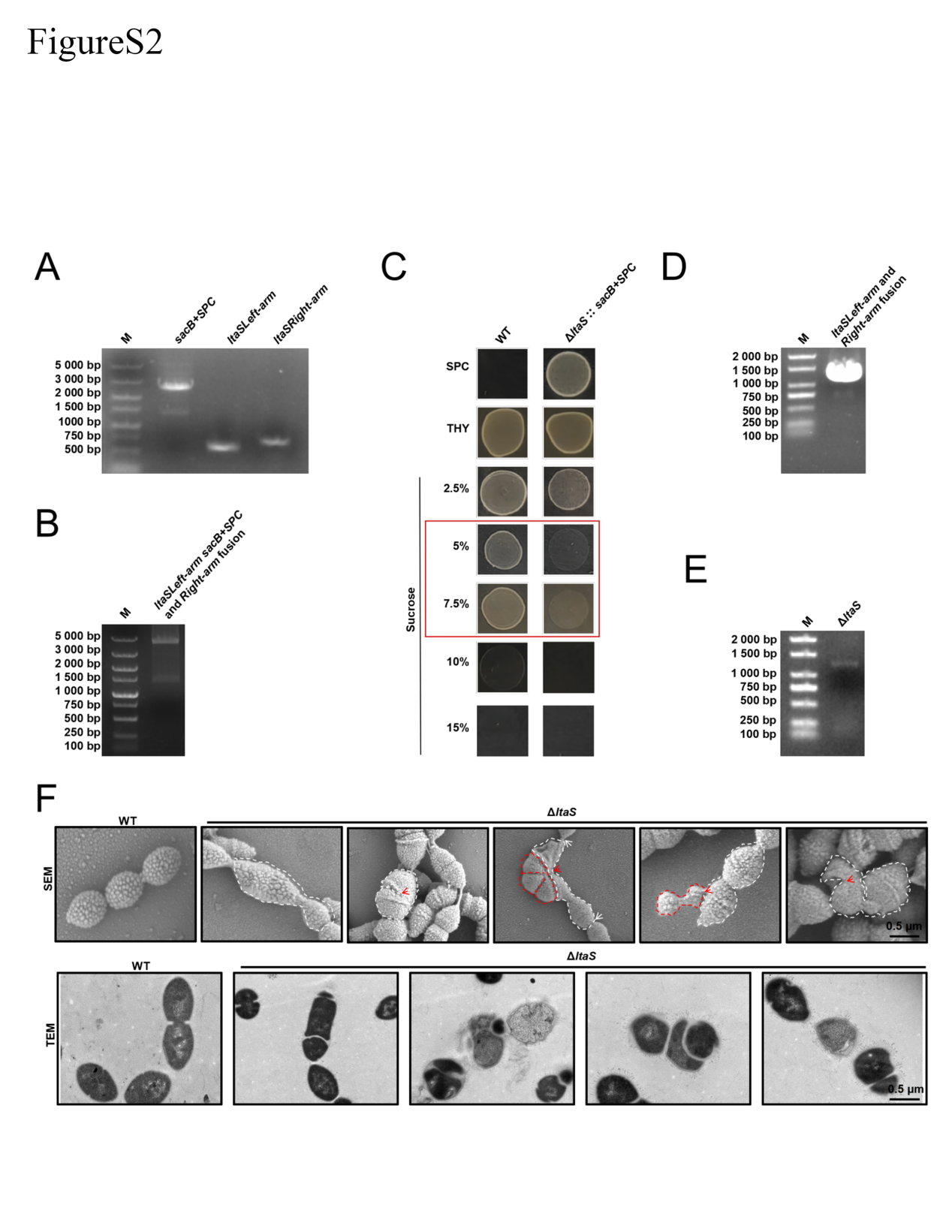
**

**Fig. S2 The construction of Δ*ltaS*.** (A) PCR amplification of the target gene fragments. (B) Fusion of *ltaS*Left-arm, *sacB*+*SPC*, and *ltaS*Right-arm gene fragments. (C) Measurement of sucrose sensitivity of *S. suis* *sacB* inserted strain. (D) Fusion of *ltaS*Left-arm and *ltaS*Right-arm gene fragments. (E) PCR identification of the ZY05719 *ltaS* gene deletion strain. (F) Observation of morphology of the WT SS2 and Δ*ltaS* by SEM and TEM. White dotted line indicates normal morphology of the Δ*ltaS.* Red dotted line and red arrow indicate morphology change of the Δ*ltaS*. Scale bar, 0.5 μm.

**
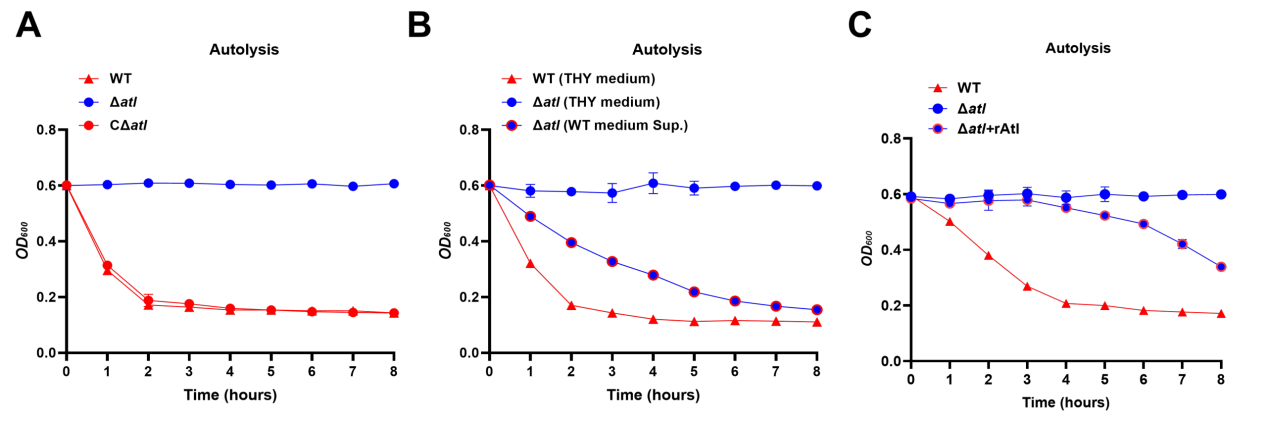
**

**Fig. S3** Autolytic activity of WT SS2 and Δ*atl*. Bacteria were resuspended in 0.05% Triton X-100 and incubated at 37℃. (A) Lytic activity of the WT SS2, Δ*atl*, and CΔ*atl* were monitored at OD_600_. (B) Lytic activity of the WT SS2, Δ*atl*, and Δ*atl* with addition of WT SS2 culture supernatant were monitored at OD_600_. **(**C) Lytic activity of the WT SS2, Δ*atl*, and Δ*atl* supplemented with rAtl were monitored at OD_600_. Data are presented as means ± SD.

**
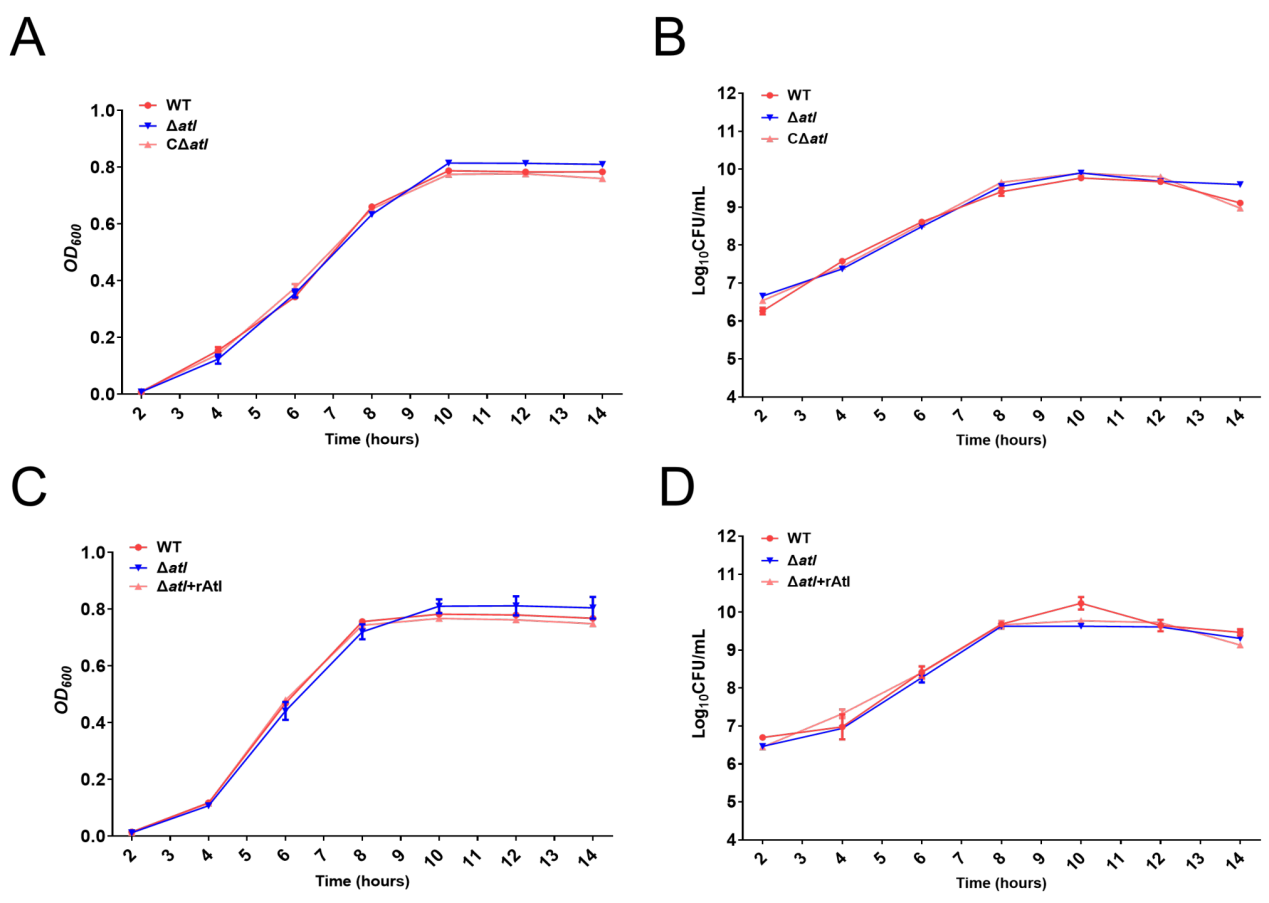
**

**Fig. S4** A growth curve was established for SS2. (A and B) The OD_600_ and CFU of WT SS2, Δ*atl*, and CΔ*atl* are shown in parallel time point. Data shown are means ± SD. (C and D) The OD_600_ and CFU of WT SS2, Δ*atl*, and Δ*atl* supplemented with rAtl are shown in parallel time point. Data shown are means ± SD.


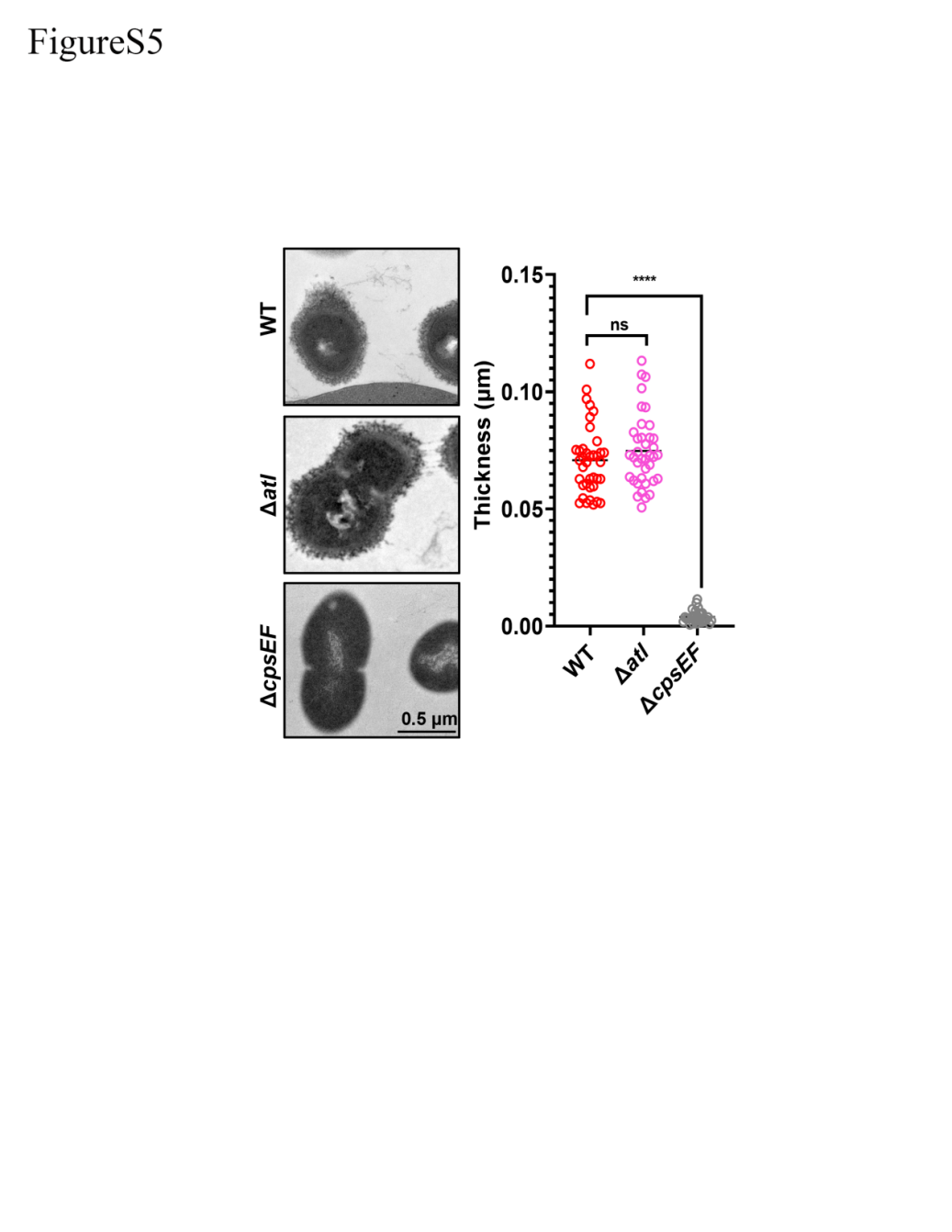


**Fig. S5** Observation of capsular morphology of SS2. The WT SS2, Δ*atl*, and Δ*cpsEF* capsular morphology were observed by TEM. Scale bar, 0.5 μm. Determination and analysis of capsule thickness of the WT SS2, Δ*atl*, and Δ*cpsEF* are shown on the right. Data are presented as means ± SD. **** *P* < 0.0001; ns, not significant (one-way ANOVA).


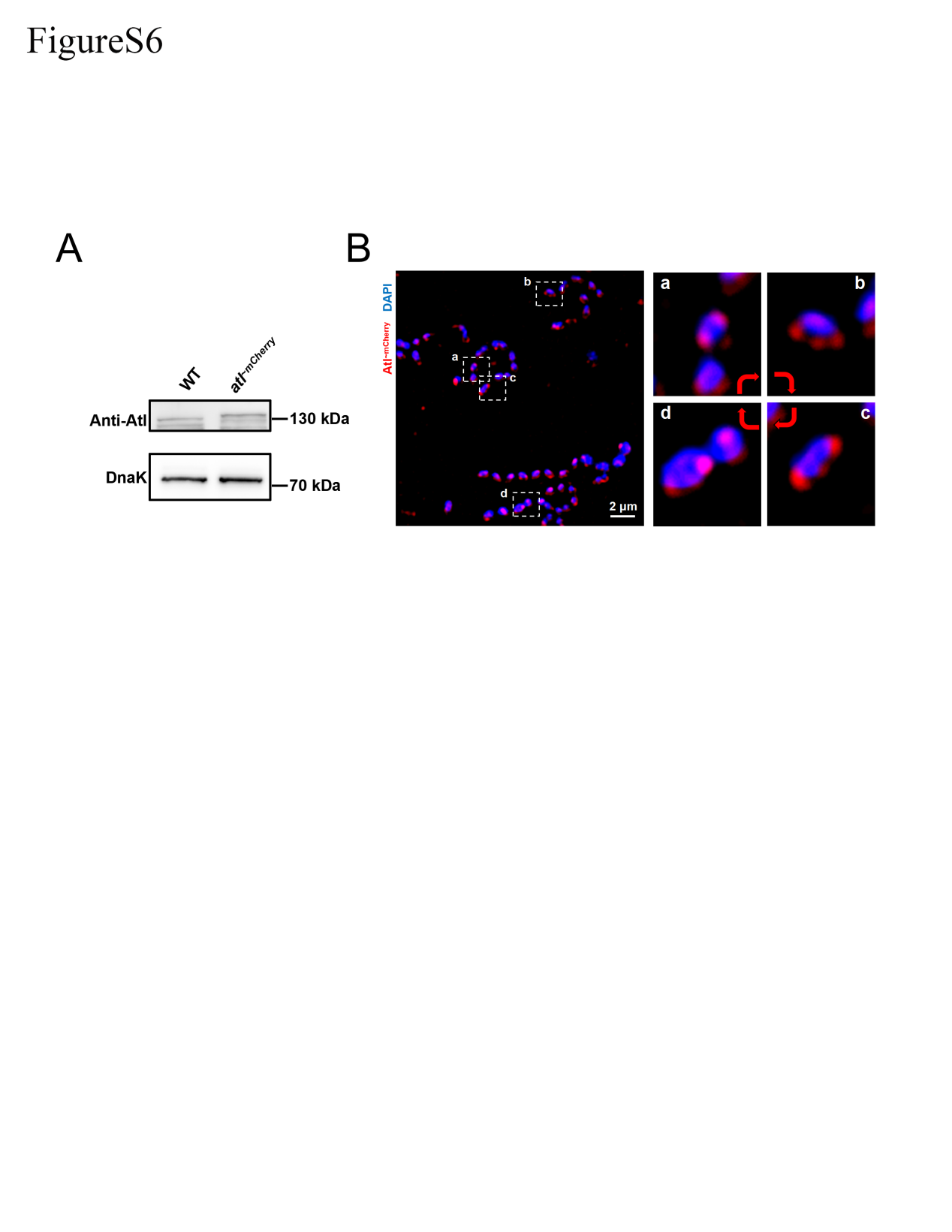


**Fig. S6** The distribution of Atl labeled by mCherry in SS2. **(**A) The presence of Atl in the SS2 was confirmed through Western blot analysis. DnaK was used as a loading control. (B) Confocal images of bacteria. The Atl was labeled by protein fusions and examined by fluorescence microscopy. The Atl labeled by mCherry (red), and nuclei of SS2 were stained with DAPI (blue). Scale bar, 2 μm.

**Table S1. The sequence of primers for construction of SS2 mutant strains and the plasmid construction.**

| **Primer name** | **Sequences（5**' **- 3**'**）** |
| --- | --- |
| *SacB*+*SPC* F | GGATAATGCTGAAAACTCCTT |
| *SacB*+*SPC* R | AATCTGATTACCAATTAGAATGAATAT |
| *ltaS* *Left-arm* F | GCTCCTCTTACTTCTGG |
| *ltaS* *Left-arm* R1 | AAGGAGTTTTCAGCATTATCCAATAAAACCTCATG |
| *ltaS* *Left-arm* R2 | AATAAAACCTCATGGTCCTTTCTAAGCCTTTTATAGTGTAAATCTG |
| *ltaS* *Right-arm* F2 | CAGATTTACACTATAAAAGGCTTAGA |
| *atl^COOH^* F | TGCATTGTCTGTTTCAAA |
| *atl^COOH^* R | AGCTTCTCCACCAGATCCCTTTCCCTCAACAGCACC |
| *atl^Bsp^* F | GATGTTCGTGTTACTAATGTTTCATC |
| *atl^Bsp^* R | AGCTTCTCCACCAGATCCTGTGTAGGATCGCTGTGCCG |
| *atl^COOH^-mCherry* F  /*atl^Bsp^-mCherry* F | GGATCTGGTGGAGAAGCTGCAGCTAAAGGATCAATGGTGAGCAAGGGCGAG |
| *mCherry* R | AAGGAGTTTTCAGCATTATCCCTACTTGTACAGCTCGTCCATGC |
| *atl* *Right-arm* F | ATATTCATTCTAATTGGTAATCAGATTATTGCAGGTACAGGTATTG |
| *atl* *Right-arm* R | CCCTTTAGTGATTTGGTC |
| pColdI-*atln* F | GAGCTCGGTACCCTCGAG*GGATCC*GTAAATGCCCCATCTAGTCA |
| pColdI-*atln* R | TCTAGACTGCAGGTCGAC*AAGCTT*CTAACTTCTTGTCAGCGAATT |
| pColdI-*atl^Bsp^* F | GAGCTCGGTACCCTCGAG*GGATCC*TCTTCAAATACGATTGCAGG |
| pColdI-*atl^Bsp^* R | TCTAGACTGCAGGTCGAC*AAGCTT*CTACGACGGATTTGATAAATGAG |
| pColdI-*atl^COOH^* F | AGCTCGGTACCCTCGAG*GGATCC*ACAGTCTATATTGACCCAGG |
| pColdI-*atl^COOH^* R | CTAGACTGCAGGTC*G*AC*AAGCTT*ATTCATTACACCTTGTG |
